# Differential effects of cannabidiol on inhibitory avoidance memory reconsolidation and consolidation are associated with distinct hippocampal proBDNF and BDNF signaling profiles

**DOI:** 10.3389/fnins.2026.1915561

**Published:** 2026-08-17

**Authors:** Wen Dai, Wan-Ling Huang, Sherry Shu-Jung Hu

**Affiliations:** Cannabinoid Signaling Laboratory, Department of Psychology, National Cheng Kung University, Tainan, Taiwan

**Keywords:** anisomycin, BDNF, cannabidiol, inhibitory avoidance, memory consolidation, memory reconsolidation, mice, proBDNF

## Abstract

**Introduction:**

Cannabidiol (CBD), a major non-psychoactive constituent of *Cannabis sativa*, exerts pleiotropic effects, including antipsychotic, anxiolytic, and neuroprotective properties, with low toxicity and minimal euphoric effects. Previous studies have demonstrated that CBD alleviates memory impairments associated with pathological conditions such as seizures, reserpine exposure, pneumococcal meningitis, and sepsis. However, its effects on memory processes, particularly inhibitory avoidance (IA) memory reconsolidation and consolidation, remain incompletely understood and inconsistently reported. Therefore, this study investigated the effects of CBD on IA memory reconsolidation and consolidation in mice and explored the underlying neurotrophic mechanisms.

**Methods:**

Mice were subjected to the IA task and administered CBD at different doses during either the reconsolidation or consolidation phase. To examine the involvement of tropomyosin receptor kinase (Trk) signaling, the Trk receptor antagonist K252a was co-administered with CBD. In addition, anisomycin (ANI) was used to induce impairments in IA memory consolidation. Hippocampal levels of pro-brain-derived neurotrophic factor (proBDNF) and mature brain-derived neurotrophic factor (BDNF) were measured to investigate the molecular mechanisms underlying CBD-mediated effects.

**Results:**

CBD impaired IA memory reconsolidation in a dose-dependent manner, and this effect was abolished by K252a. During reconsolidation, CBD selectively reduced hippocampal proBDNF levels without altering mature BDNF expression. In contrast, the same dose of CBD did not affect IA memory consolidation under baseline conditions. However, CBD attenuated ANI-induced impairments in IA memory consolidation, and this protective effect was also blocked by K252a. Biochemical analyses further showed that ANI treatment reduced hippocampal mature BDNF levels, whereas CBD administration prevented this reduction.

**Discussion:**

These findings demonstrate that CBD differentially modulates IA memory reconsolidation and consolidation through distinct neurotrophic mechanisms. Specifically, the CBD-induced impairment of memory reconsolidation was associated with reduced hippocampal proBDNF levels, whereas the protective effect of CBD against ANI-induced deficits in memory consolidation was accompanied by restoration of mature BDNF expression relative to the vehicle control group. Collectively, these findings provide novel mechanistic insights into the bidirectional effects of CBD on memory processes and underscore the importance of carefully evaluating its therapeutic potential and associated neurobiological mechanisms in the context of memory-related disorders.

## 1 Introduction

Cannabidiol (CBD) is a pleiotropic compound that targets multiple pharmacological systems within the central nervous system (CNS). As one of the most abundant phytocannabinoids in *Cannabis sativa*, CBD shares structural similarity and therapeutic potential with Δ^9^-tetrahydrocannabinol (THC). However, in contrast to THC, CBD exhibits antipsychotic and anxiolytic properties with minimal hedonic effects (Babalonis et al., 2017; Hurd et al., 2015; Pintori et al., 2023; Stella, 2023). Moreover, CBD demonstrates low toxicity and is generally better tolerated than THC (Hurd, 2017; Martin-Santos et al., 2012). These differences are largely attributed to CBD’s low affinity for cannabinoid CB_1_ and CB_2_ receptors (Pertwee, 2008). Instead of acting as a direct agonist, CBD interacts with allosteric binding sites on CB_1_ and CB_2_ receptors, functioning as an inverse agonist or antagonist (Laprairie et al., 2015; Martínez-Pinilla et al., 2017). In addition to its actions on the endocannabinoid system, CBD modulates neuroplasticity associated with emotional regulation through interactions with serotonergic signaling and brain-derived neurotrophic factor (BDNF)–tropomyosin receptor kinase B (TrkB) pathways. Specifically, CBD acts as an orthosteric agonist and positive allosteric modulator at 5-HT_1A_ receptors, while serving as a negative allosteric modulator at 5-HT_3A_ receptors (Russo et al., 2005; Vitale et al., 2021). Through these serotonergic mechanisms, CBD may indirectly facilitate BDNF–TrkB signaling, thereby contributing to its neuromodulatory and therapeutic effects (Guldager et al., 2024).

The versatile role of CBD in affective and cognitive functions, particularly in the modulation of aversive memory processing, has been well documented (Blessing et al., 2015; Lee et al., 2017; Papagianni and Stevenson, 2019; Wright et al., 2020). Preclinical studies have demonstrated that CBD impairs both the acquisition (Levin et al., 2012) and consolidation (Stern et al., 2017) of memory in the contextual fear conditioning (CFC) paradigm. However, targeting these early stages of memory processing may have limited clinical applicability, as it is not feasible to predict whether a traumatic event will subsequently result in a pathological condition. In contrast, interventions targeting later stages of memory processing may offer greater therapeutic potential. CBD has been shown to impair the reconsolidation (Gazarini et al., 2014; Stern et al., 2012, 2015) and retrieval (Lemos et al., 2010) of CFC memory, while facilitating extinction (Bitencourt et al., 2008; Do Monte et al., 2013). These processes are particularly relevant to exposure-based psychotherapies, which rely on the reactivation of consolidated memories. Upon re-exposure, previously consolidated memories enter a transiently labile state, during which they may undergo reconsolidation or extinction and thus become susceptible to pharmacological modulation (Nader et al., 2000; Suzuki et al., 2004). Mechanistically, the impairing effects of CBD on CFC memory have been primarily attributed to the involvement of 5-HT_1A_ and CB_1_ receptors (Bitencourt et al., 2008; Do Monte et al., 2013).

The CFC and inhibitory avoidance (IA) tasks are widely used paradigms for studying aversive memory, yet they differ in behavioral structure, neural circuitry, and functional relevance. In the CFC task, subjects learn an association between a neutral context (conditioned stimulus, CS) and an aversive foot-shock (unconditioned stimulus, US), such that re-exposure to the context elicits a conditioned fear response. In contrast, the IA task extends beyond classical conditioning by incorporating an operant component, in which animals voluntarily enter a dark compartment where they receive foot-shock and subsequently learn to avoid it, thereby modeling active coping behavior (Liang, 2009). Consistent with this distinction, CFC primarily reflects passive fear learning, whereas IA more closely resembles adaptive avoidance strategies relevant to human stress coping. At the neural level, the amygdala is required for CFC acquisition but is differentially involved in IA, whereas the dorsal hippocampus and medial prefrontal cortex are engaged in both paradigms but contribute more prominently to contextual and integrative processing in IA, including its interaction with the nucleus accumbens. Moreover, pharmacological and circuit-level studies indicate that IA memory consolidation depends on a broader hippocampal–prefrontal–striatal network, whereas CFC relies on a more amygdala-centered circuit (Liang, 2009). Together, these findings suggest that IA engages more complex associative and decision-making processes than CFC, making it a valuable model for studying adaptive fear memory and related neuropsychiatric disorders.

To date, however, no studies have directly examined the effects of CBD on memory reconsolidation in the IA task. Existing evidence indicates that CBD either exerts no significant effect on IA memory consolidation (Fagherazzi et al., 2012) or enhances IA memory acquisition, consolidation, and retrieval (Kruk-Slomka and Biala, 2021). Notably, the same group reported that subeffective doses of CBD can reverse memory impairments induced by MK-801 and scopolamine in the IA paradigm (Kruk-Slomka and Biala, 2021; Kruk-Slomka et al., 2024). Collectively, these findings suggest that the direction and magnitude of CBD’s modulatory effects on aversive memory are context-dependent, influenced by factors such as the behavioral paradigm employed, dosing regimen, specific phase of memory processing, and the underlying neurobiological mechanisms engaged.

Notably, memory reconsolidation does not merely recapitulate the processes underlying the consolidation of newly acquired information. Although these processes share certain overlapping cellular and molecular mechanisms, memory reconsolidation engages distinct neural circuits and is governed by different temporal dynamics (Alberini, 2005; Debiec et al., 2002; McKenzie and Eichenbaum, 2011; Miller and Sweatt, 2006; Riccio and Cullen, 2012). A series of studies by Lee and colleagues further substantiate this dissociation, demonstrating that the consolidation and reconsolidation of CFC memories within the rat hippocampus are mediated by independent molecular mechanisms and divergent cellular pathways. Specifically, consolidation has been shown to depend on BDNF, but not on zif268, whereas reconsolidation critically requires zif268 while being independent of BDNF (Lee et al., 2004). Moreover, these two processes are supported by distinct intracellular signaling cascades: consolidation involves an NMDA receptor–ERK1–BDNF pathway, whereas reconsolidation engages an NMDA receptor–IKKα–zif268 signaling cascade (Lee and Hynds, 2013). Together, these findings highlight a clear mechanistic dissociation between consolidation and reconsolidation at both the molecular and circuit levels.

Emerging evidence suggests that CBD interacts with serotonergic signaling and neurotrophin-related pathways, including the BDNF–TrkB axis, to modulate emotion-related neuroplasticity (Guldager et al., 2024). The hippocampus is particularly enriched in BDNF, a key neurotrophin critically involved in synaptic plasticity, learning, and memory (Murer et al., 2001). Consistent with this, hippocampal BDNF expression is significantly upregulated during spatial learning tasks (Mizuno et al., 2000). Pharmacological manipulations of this pathway have been shown to exert robust effects on memory processes: exogenous BDNF enhances both consolidation and reconsolidation of IA memory, whereas blockade or disruption of BDNF signaling impairs memory formation and plasticity (Bekinschtein et al., 2008b; Lu et al., 2008; Minichiello, 2009). Moreover, intrahippocampal administration of recombinant BDNF has been demonstrated to rescue anisomycin (ANI)-induced impairments in IA memory, further supporting a causal role of BDNF signaling in hippocampus-dependent memory modulation (Bekinschtein et al., 2008b). With respect to CBD, accumulating evidence indicates that its behavioral effects are closely associated with neuroplasticity-related signaling, including the BDNF–TrkB pathway. Acute CBD administration has been reported to reduce depressive- and anxiety-like behaviors in association with increased hippocampal BDNF expression (Sartim et al., 2018). In contrast, repeated CBD exposure has been shown to alter fear-related behavior accompanied by reductions in hippocampal BDNF and TrkB protein levels (ElBatsh et al., 2012), suggesting that CBD-induced behavioral outcomes may depend on bidirectional modulation of this pathway. Importantly, pharmacological evidence further supports a functional interaction between CBD-induced effects and TrkB signaling, as blockade of TrkB-mediated signaling has been shown to attenuate neuroplasticity-dependent behavioral responses in related paradigms, indicating that intact BDNF–TrkB signaling is necessary for full expression of CBD-associated neurobehavioral effects (Guldager et al., 2024). Collectively, these findings provide a mechanistic basis for focusing on the BDNF–TrkB pathway in the present study, as this signaling cascade plays a central role in hippocampus-dependent memory processing and may critically mediate the cognitive effects of CBD.

A synthesis of the aforementioned literature indicates that CBD modulates multiple phases of CFC memory. However, its effects on the consolidation of IA memory remain inconclusive, and its role in IA memory reconsolidation has yet to be systematically elucidated. The present study was therefore designed to investigate the effects of CBD on both the consolidation and reconsolidation of IA memory, with particular emphasis on the involvement of hippocampal BDNF–TrkB signaling. In the first experiment, the impact of CBD on IA memory reconsolidation was examined. In the second experiment, the effect of CBD on IA memory consolidation was assessed. Furthermore, the potential of CBD to reverse ANI-induced memory impairment was evaluated. To further elucidate the underlying molecular mechanisms, western blot analysis was performed to quantify hippocampal BDNF protein levels and to determine their role in mediating the effects of CBD on IA memory consolidation and reconsolidation.

## 2 Materials and methods

### 2.1 Animals

Male C57BL/6J mice, 8–14 weeks old, were purchased from the National Laboratory Animal Center (Taipei, Taiwan) and the Laboratory Animal Center of National Cheng Kung University (NCKU) College of Medicine (Tainan, Taiwan). The vivarium is a temperature (21–23°C) and humidity (60%–70%)-controlled colony room under a 12-h light/dark cycle (light on at 0700 h). The mice were group-housed (5 per cage) with *ad libitum* access to food and water. Prior to each behavioral experiment, all cages were transported to the behavioral testing room using a cart and allowed to habituate to the testing environment for at least 1 h before the commencement of testing. All experimental procedures conformed to the Guide for the Care and Use of Laboratory Animals (NIH Publications No. 80-23 in USA) and the ARRIVE guidelines 2.0 and were approved by the Institutional Animal Care and Use Committee of NCKU College of Medicine (NCKU IACUC Approval No: 107229). The NCKU Laboratory Animal Center was accredited by the Association for Assessment and Accreditation of Laboratory Animal Care International (AAALAC).

### 2.2 Drugs

CBD was purchased from Hansu Company (Beijing, China). ANI was purchased from Fermentek Ltd., (Jerusalem, Israel). K252a was purchased from Sigma-Aldrich (MO, USA). Drugs and vehicles (Veh) were delivered in a volume of 10 ml/kg body weight for intraperitoneal (i.p.) or subcutaneous (s.c.) injection in mice. The Veh for CBD was ethanol/cremophor/saline (1:1:18; v/v/v). The high-concentration stock of ANI was dissolved in 1M HCl. The ANI solutions were prepared in saline and then adjusted pH to 7.4 with 1M NaOH; saline was used as its Veh. The Veh for K252a was saline containing 2% dimethyl sulfoxide (DMSO). The doses for systemic administration of CBD (5, 10, 30 mg/kg, i.p.), ANI (50, 150 mg/kg, s.c.), and K252a (5 μg/kg, i.p.) were adopted from previous studies (Autry et al., 2011; Flood et al., 1973; Huang et al., 2021; Kruk-Slomka and Biala, 2021; Liao et al., 2016; Monleón Verdú et al., 2008; Murillo-Rodríguez et al., 2018; Zhang et al., 2011). All drugs were freshly prepared before use.

### 2.3 Inhibitory avoidance (IA) task

The single-trial step-through IA task was used, where mice learned to associate a location in the IA apparatus (a dark compartment) with an aversive stimulus (footshock), as described in our previous studies (Hsiung et al., 2020; Huang et al., 2021). The IA apparatus is a custom-made acrylic box consisting of two compartments: the light and dark compartments. The light compartment (17.5 × 16 × 26 cm) was illuminated by a lamp (900 ∼ 1050 lm), whereas the dark compartment (24 × 16 × 26 cm) was made with a floor of stainless steel bars. A movable sliding door controlled the passages between the light and dark compartments. An electrical shock generator (SINGA, Taiwan) was connected to the floor bars of the dark compartment. The IA chambers were wipe-cleaned with a 70% isopropyl alcohol-rinsed paper towel after each mouse finished the experiments.

#### 2.3.1 Memory reconsolidation

**FIGURE 1:**
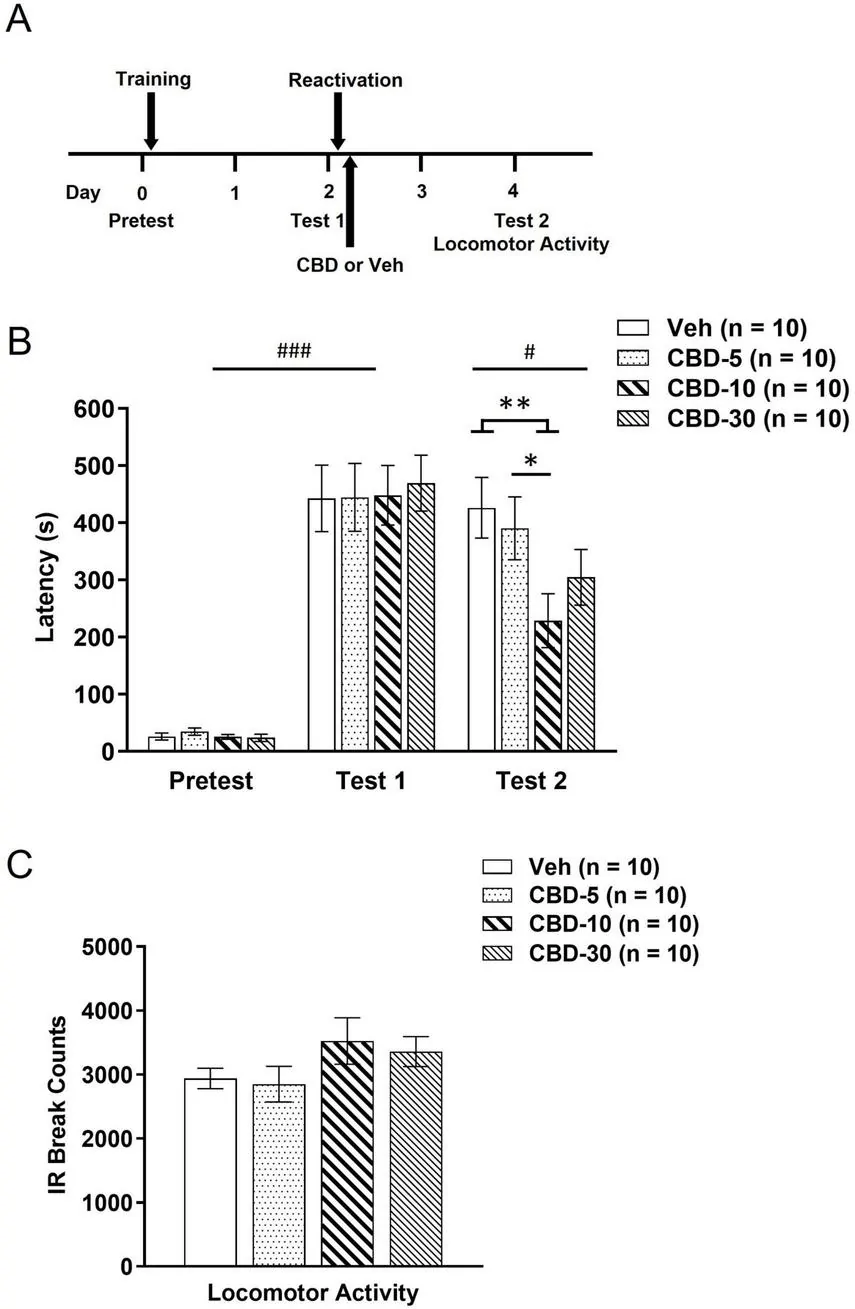
Cannabidiol (CBD) impaired IA memory reconsolidation. **(A)** Experimental timeline illustrating the effects of CBD on IA memory reconsolidation. Mice were randomly assigned into four groups (Veh or CBD at 5, 10, or 30 mg/kg; *n* = 10 per group). **(B)** A two-way repeated-measures ANOVA revealed that all mice successfully acquired IA memory during Test 1 (time, F_1,36_ = 251.195, ^###^***p*** < 0.001). In contrast, a one-way ANOVA revealed a significant group effect in Test 2 (F_3,36_ = 3.014, ^#^*p* < 0.05). *Post hoc* analyses revealed that mice treated with CBD at 10 mg/kg exhibited significantly reduced IA memory performance compared with both the Veh group (**p < 0.01) and the CBD 5 mg/kg group (*p < 0.05). No significant differences were observed for the 30 mg/kg group relative to controls. **(C)** CBD administration did not affect locomotor activity in mice (one-way ANOVA: F_3,36_ = 1.459, *p* > 0.05).

In the memory reconsolidation procedure of the IA task, the mouse first underwent IA training. The mouse was placed at the far end of the light compartment facing away from the door. As the mouse turned around, the sliding door opened. After the mouse entered the dark compartment with its four paws, the door was closed, and an inescapable footshock (0.1 or 0.2 mA) was delivered for 3 s after a 3-s delay. After IA training, the mouse was left in the dark compartment for another 10 s, then removed and returned to its home cage. The step-through latency of the dark compartment entry in the training was used as a baseline in the pretest (Figure 1A). In Test 1 on day 2, each mouse was placed into the light compartment and the step-through latency of the dark compartment entry was recorded as the IA memory retention score. Test 1 was terminated after 600 s if the mouse did not step into the dark compartment. Eventually, the mouse was forced with a black acrylic plate held by researchers to enter the dark compartment, and the door was closed. For memory reactivation, the mouse was left in the dark compartment for an additional 10 s without any footshock, then removed and returned to the home cage (Figure 1A). In Test 2 on day 4, the mouse was again placed into the light compartment and the step-through latency of the dark compartment entry was recorded as the IA memory retention score (Figure 1A). The 2-day period between the IA training, Test 1, and Test 2 was adopted from a prior study (Milekic and Alberini, 2002).

**FIGURE 2:**
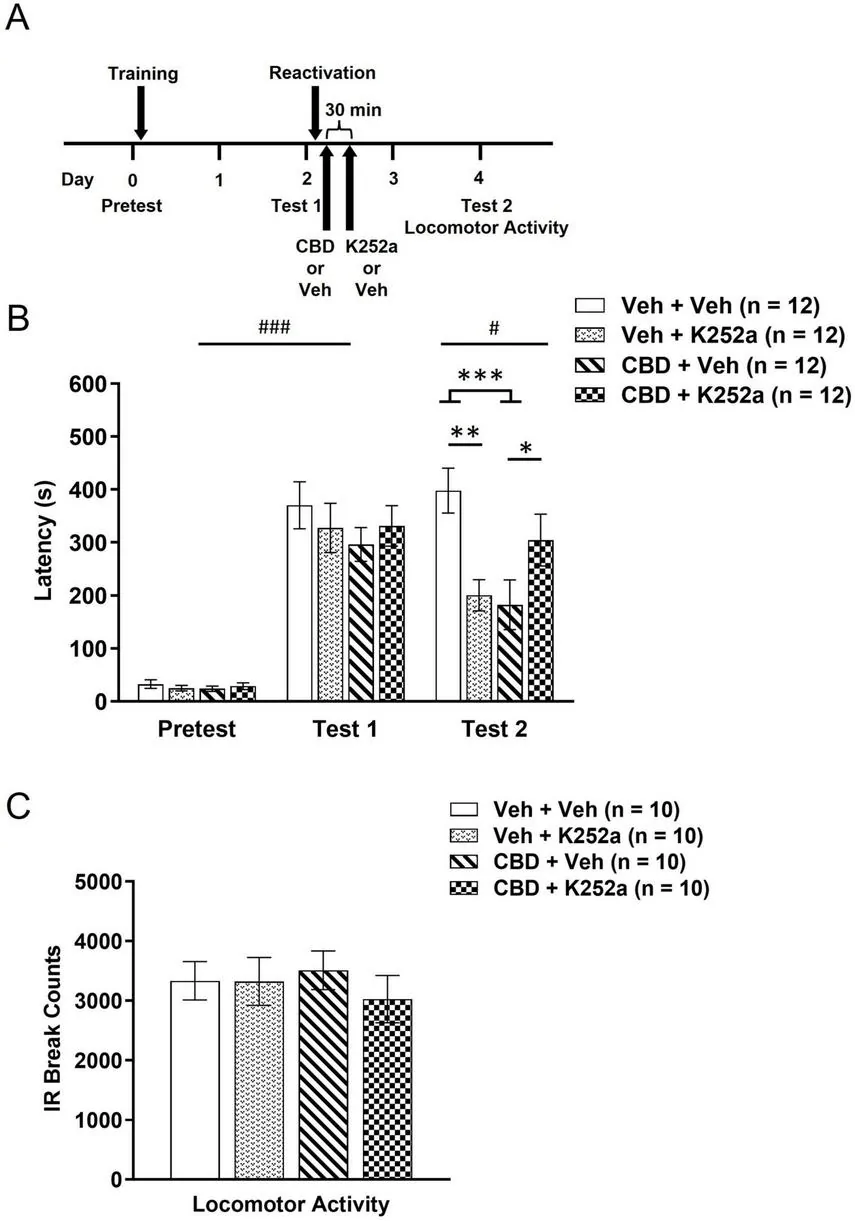
K252a reversed CBD’s impairing effect on IA memory reconsolidation. **(A)** Experimental timeline illustrating the effects of K252a (5 μg/kg) on CBD-induced impairment of IA memory reconsolidation following CBD administration (10 mg/kg). Mice were randomly assigned to four groups (*n* = 12 per group): Veh + Veh, Veh + K252a, CBD + Veh, and CBD + K252a. **(B)** A two-way repeated-measures ANOVA revealed that all mice successfully acquired IA memory in Test 1 (time, F_1,44_ = 220.784, ^###^*p* < 0.001). In Test 2, a one-way ANOVA revealed a significant group effect (F_3,44_ = 5.522, ^#^*p* < 0.05). *Post hoc* analyses revealed that both the Veh + K252a and CBD + Veh groups exhibited significant impairments in IA memory reconsolidation (**p < 0.01; ***p < 0.001), indicating that administration of K252a or CBD alone impaired IA memory reconsolidation. Notably, the CBD + K252a group showed significantly greater IA memory retention compared with the CBD + Veh group (*p < 0.05), indicating that K252a reversed the impairing effect of CBD on IA memory reconsolidation. **(C)** Administration of CBD and K252a, either alone or in combination, did not affect locomotor activity in mice (one-way ANOVA: F_3,36_ = 0.309, *p* > 0.05).

A cohort of mice was used to investigate the effect of CBD on IA memory reconsolidation. CBD or Veh was administered immediately after memory reactivation (Figure 1A). The mice were randomly divided into four groups (*n* = 10 per group): vehicle (Veh), 5 mg/kg CBD (CBD-5), 10 mg/kg CBD (CBD-10), and 30 mg/kg CBD (CBD-30). Another cohort of mice was used to investigate whether K252a (5 μg/kg) could reverse CBD’s memory-impairing effect (10 mg/kg) on IA memory reconsolidation. CBD or Veh was administered immediately after memory reactivation, and K252a or Veh was injected 30 min after CBD was administered (Figure 2A). The mice were randomly assigned into four groups (*n* = 12 per group): Veh + Veh, Veh + K252a, CBD + Veh, and CBD + K252a. At the end of Test 2, all groups of mice (*n* = 10 per group) were randomly selected to examine their locomotor activity.

#### 2.3.2 Memory consolidation

In the memory consolidation procedure of the IA task, the mouse underwent IA training. In brief, the mouse was placed at the far end of the light compartment, and the sliding door was opened when the mouse turned around. After the mouse entered the dark compartment with its four paws, the door was closed, and an inescapable footshock (0.2 mA) was delivered for 3 s after a 3-s delay. After IA training, the mouse was left in the dark compartment for another 10 s, then removed and returned to its home cage. The step-through latency of the dark compartment entry in the training was used as a baseline in the pretest (Figure 4A). In the test on day 2, each mouse was placed into the light compartment and the step-through latency of the dark compartment entry was recorded as the IA memory retention score. The test was terminated after 600 s if the mouse did not step into the dark compartment. The 2-day period between the IA training and test was adopted from a prior study (Milekic and Alberini, 2002).

**FIGURE 3:**
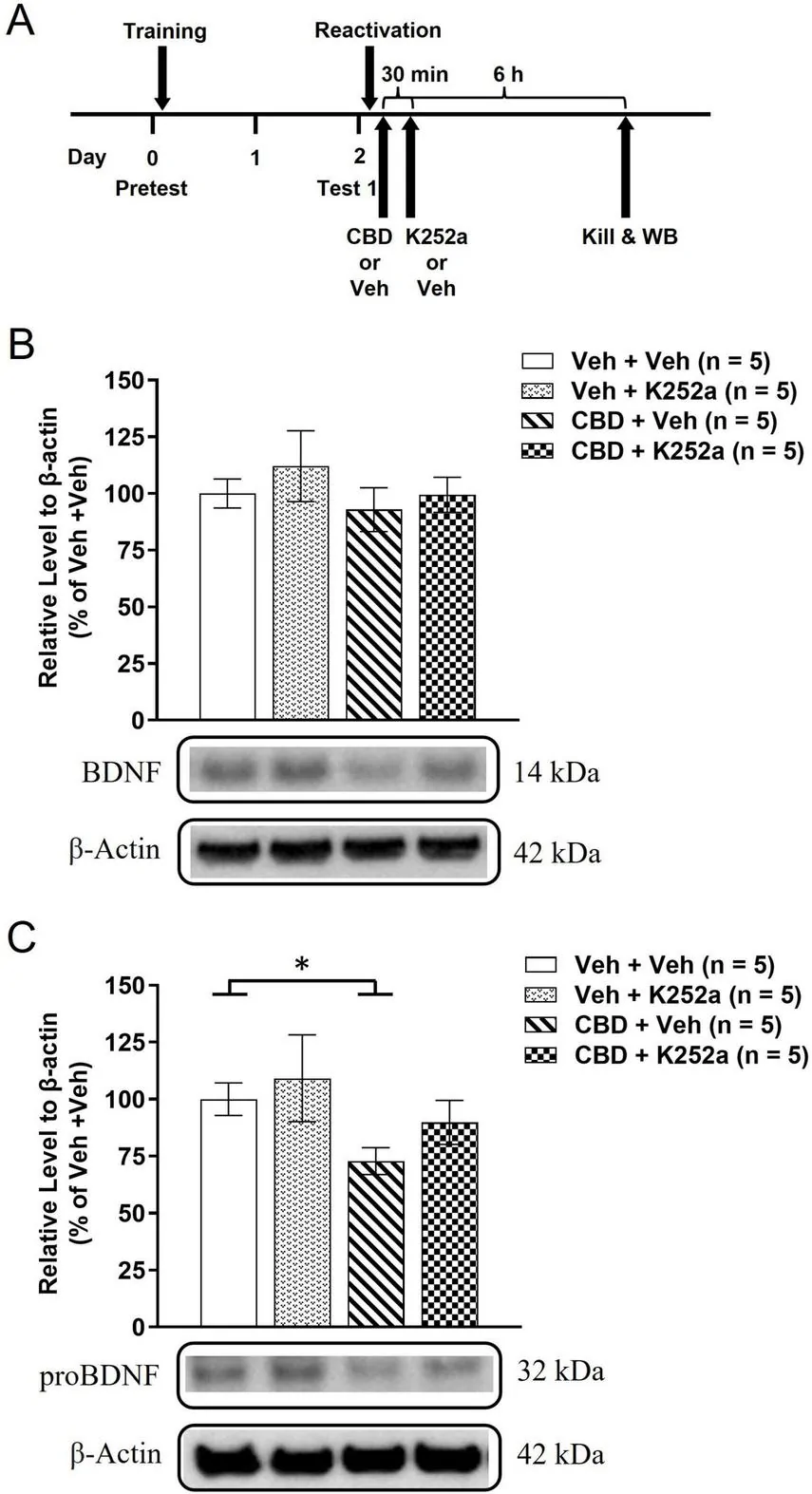
Cannabidiol (CBD) decreased hippocampal proBDNF protein levels at 6 h after memory reactivation. **(A)** Experimental timeline illustrating the effects of CBD (10 mg/kg), followed by injection of K252a (5 μg/kg) 30 min later, on hippocampal BDNF protein expression at 6 h after memory reactivation. Mice were randomly assigned to four groups (*n* = 5 per group): Veh + Veh, Veh + K252a, CBD + Veh, and CBD + K252a. **(B)** A one-way ANOVA revealed that no significant differences among the four groups in hippocampal mature BDNF protein (14 kDa) levels at 6 h after memory reactivation (F_3,16_ = 0.582, *p* > 0.05). **(C)** *A priori* comparisons using independent *t*-tests revealed that CBD alone (CBD + Veh) significantly reduced hippocampal proBDNF levels relative to the Veh + Veh group (*p < 0.05). A one-way ANOVA, however, revealed that no significant group differences in proBDNF (32 kDa) levels (F_3,16_ = 1.783, *p* > 0.05). CBD + K252a group exhibited proBDNF levels that did not significantly differ from those of the Veh + Veh, Veh + K252a, or CBD + Veh groups (all *p* > 0.05). These findings suggest that CBD selectively reduces hippocampal proBDNF levels, an effect that is partially reversed by K252a administration.

**FIGURE 4:**
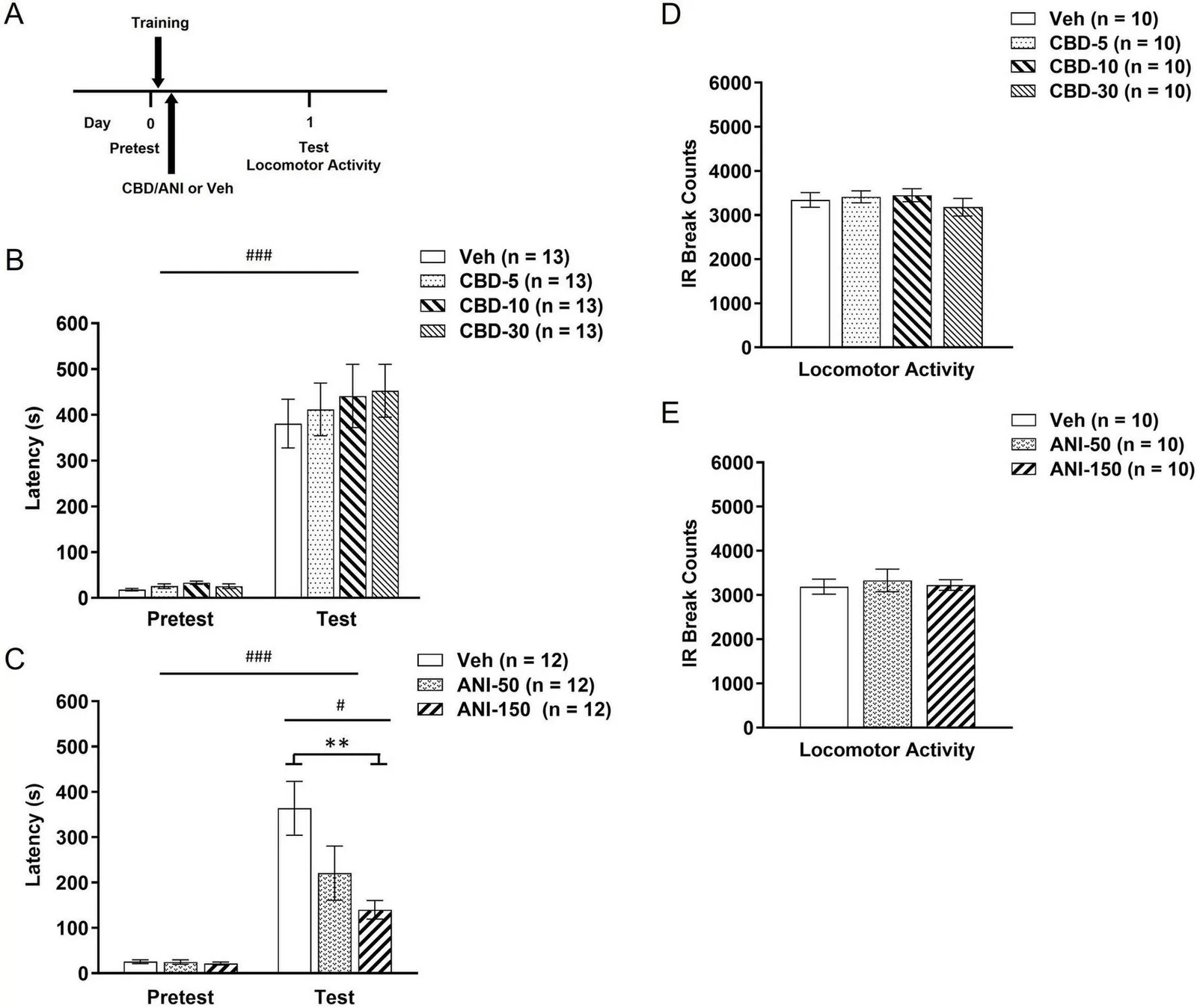
Cannabidiol (CBD), unlike ANI, did not impair IA memory consolidation. **(A)** Experimental timeline illustrating the effects of CBD (5, 10, or 30 mg/kg) and ANI (50 or 150 mg/kg) on IA memory consolidation. **(B)** Mice were randomly assigned into four groups (Veh or CBD at 5, 10, or 30 mg/kg; *n* = 13 per group). A two-way repeated-measures ANOVA revealed that all mice successfully acquired IA memory during the test session (time, F_1,48_ = 179.596, ^###^*p* < 0.001). A one-way ANOVA revealed that CBD administration did not affect IA memory consolidation (F_3,48_ = 0.29, *p* > 0.05). **(C)** Mice were randomly assigned into three groups (Veh or ANI at 50 or 150 mg/kg; *n* = 12 per group). A two-way repeated-measures ANOVA revealed that all mice successfully acquired IA memory during the test session (time, F_1,33_ = 57.223, ^###^*p* < 0.001). Analysis of simple main effects revealed a significant group difference following ANI treatment (F_2,33_ = 5.128, ^#^*p* < 0.05). *Post hoc* tests showed that administration of ANI at 150 mg/kg, but not 50 mg/kg, significantly impaired IA memory consolidation (**p < 0.01), indicating a dose-dependent impairing effect of ANI on IA memory consolidation. **(D)** CBD administration did not affect locomotor activity in mice (one-way ANOVA: F_3,36_ = 0.536, *p* > 0.05). **(E)** ANI administration did not affect locomotor activity in mice (one-way ANOVA: F_2,27_ = 0.151, *p* > 0.05).

**FIGURE 5:**
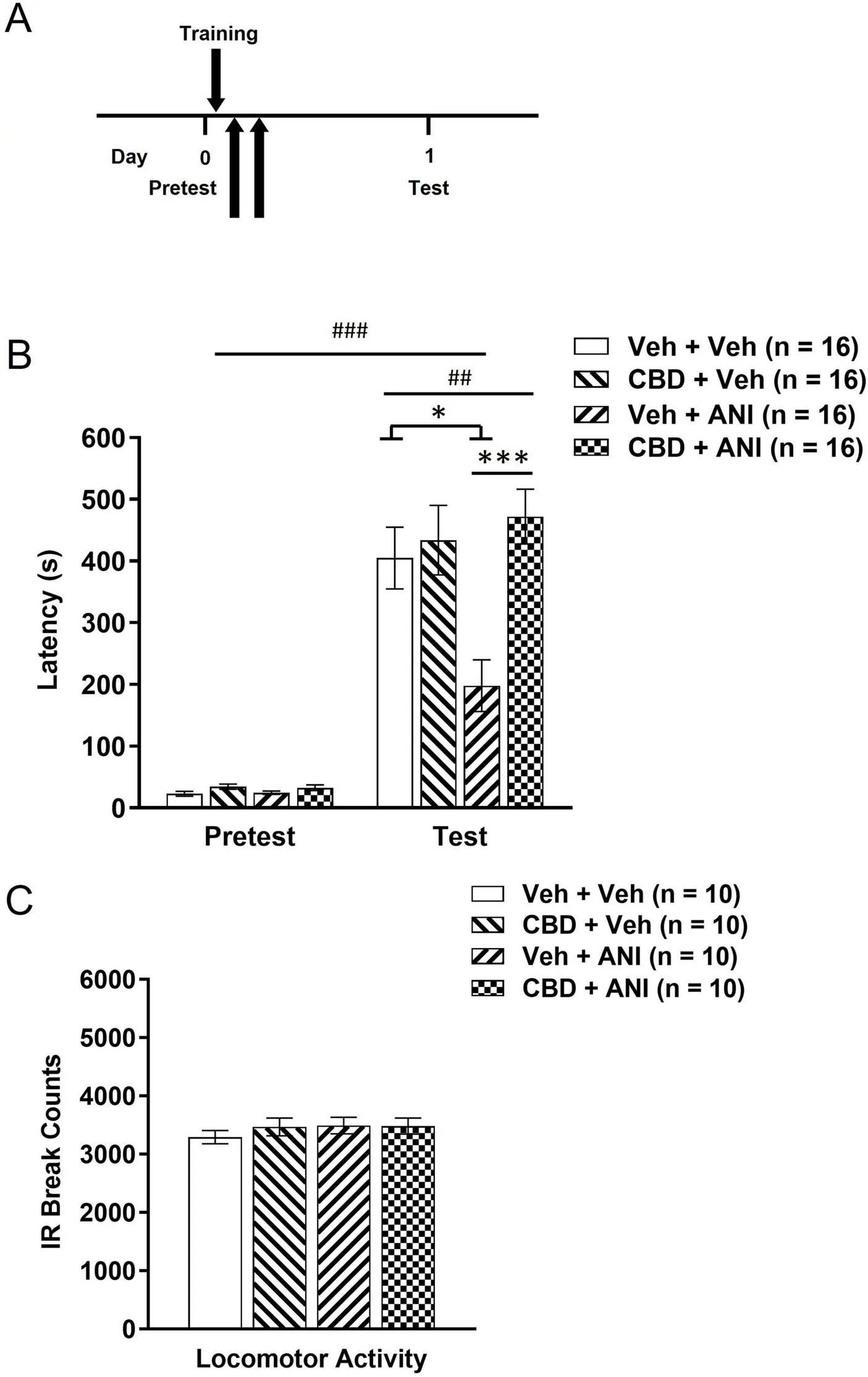
Cannabidiol (CBD) reversed ANI’s impairing effect on IA memory consolidation. **(A)** Experimental timeline illustrating the effects of CBD (10 mg/kg) on ANI (150 mg/kg)-induced impairment of IA memory consolidation. **(B)** Mice were randomly assigned to four groups (*n* = 16 per group): Veh + Veh, CBD + Veh, Veh + ANI, and CBD + ANI. A two-way repeated-measures ANOVA revealed that all mice successfully acquired IA memory during the test session (time, F_1,60_ = 211.715, ^###^*p* < 0.001). Simple main effects analysis indicated that group differences were significant only during the test phase following drug administration (F_3,60_ = 6.4, ^##^*p* < 0.01). *Post hoc* analyses revealed that the Veh + ANI group exhibited significant impairments in IA memory retention (*p < 0.05), whereas the CBD + Veh group showed intact IA memory (*p* > 0.05). Moreover, the CBD + ANI group exhibited significantly greater IA memory retention than the Veh + ANI group (***p < 0.001), indicating that CBD reversed the impairing effect of ANI on IA memory consolidation. **(C)** Administration of CBD and ANI, either alone or in combination, did not affect locomotor activity in mice (one-way ANOVA: F_3,36_ = 0.469, *p* > 0.05).

**FIGURE 6:**
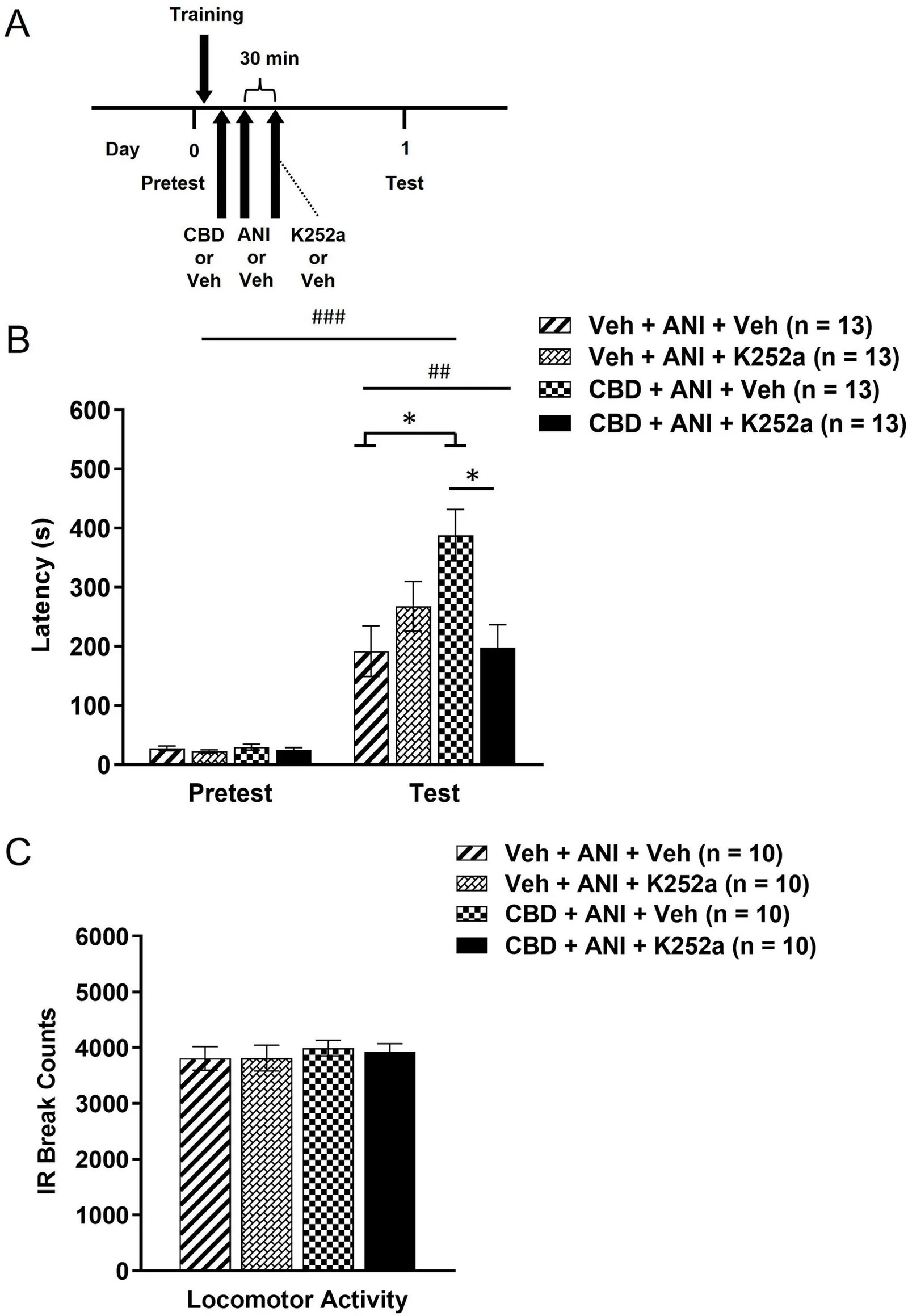
K252a blocked CBD’s rescue of ANI-induced impairment in IA memory consolidation. **(A)** Experimental timeline illustrating the effects of K252a (5 μg/kg) on the rescuing effect of CBD (10 mg/kg) against ANI (150 mg/kg)-induced impairment of IA memory consolidation. **(B)** Mice were randomly assigned to four groups (*n* = 13 per group): Veh + ANI + Veh, Veh + ANI + K252a, CBD + ANI + Veh, and CBD + ANI + K252a. A two-way repeated-measures ANOVA revealed that all mice successfully acquired IA memory during the test session (time, F_1,48_ = 138.968, ^###^*p* < 0.001). Simple main effects analysis showed that group differences were significant only during the retention test (F_3,48_ = 4.76, ^##^*p* < 0.01). *Post hoc* tests demonstrated that the CBD + ANI + Veh group exhibited significantly greater IA memory retention than the Veh + ANI + Veh group (*p < 0.05), indicating that CBD rescued the impairing effect of ANI on IA memory consolidation. In contrast, the CBD + ANI + K252a group showed significantly lower IA memory retention than the CBD + ANI + Veh group (*p < 0.05), indicating that K252a blocked the rescuing effect of CBD on ANI-induced impairment of IA memory consolidation. **(C)** Administration of CBD, ANI, and K252a, either alone or in combination, did not affect locomotor activity in mice (one-way ANOVA: F_3,36_ = 0.234, *p* > 0.05).

A cohort of mice was used to investigate the effect of CBD on IA memory consolidation. CBD or Veh was administered immediately after IA training (Figure 4A). The mice were randomly divided into four groups (*n* = 13 per group): Veh, 5 mg/kg CBD (CBD-5), 10 mg/kg CBD (CBD-10), and 30 mg/kg CBD (CBD-30). The second cohort of mice was used to investigate the effect of ANI on IA memory consolidation. ANI or Veh was administered immediately after IA training (Figure 4A). The mice were randomly assigned into three groups (*n* = 12 per group): Veh, 50 mg/kg ANI (ANI-50), and 150 mg/kg ANI (ANI-150). The third cohort of mice was used to investigate whether CBD (10 mg/kg) could reverse ANI’s memory-impairing effect (150 mg/kg) on IA memory consolidation. CBD and ANI (or their Veh) were administered consecutively after IA training (Figure 5A). The mice were randomly assigned into four groups (*n* = 16 per group): Veh + Veh, CBD + Veh, Veh + ANI, and CBD + ANI. Finally, a cohort of mice was used to investigate whether K252a (5 μg/kg) could block the rescuing effect of CBD (10 mg/kg) on ANI’s memory-impairing effect (150 mg/kg) on IA memory consolidation. CBD and ANI (or their Veh) were administered consecutively after IA training, and K252a or Veh was injected 30 min later (Figure 6A). The mice were randomly assigned into four groups (*n* = 13 per group): Veh + ANI + Veh, Veh + ANI + K252a, CBD + ANI + Veh, and CBD + ANI + K252a. At the end of the test, all groups of mice (*n* = 10 per group) were randomly selected to examine their locomotor activity.

### 2.4 Locomotor activity

The level of locomotor activity was used to evaluate whether systemic administration of CBD, ANI, and K252a would interfere with mouse motor activity and general motivation. The locomotor activity was monitored after the Test 2 of IA memory reconsolidation (48 h after drug treatments) and the test of IA memory consolidation (24 h after drug treatments). Each mouse was placed in the center of a transparent polypropylene chamber (45 × 45 × 20 cm) inside the Actitract (Panlab Harvard Apparatus, Barcelona, Spain) and allowed free navigation for a total of 15 min. Locomotor activity was defined as the infrared (IR) break count number, which is the summation of the vertical rearing and horizontal ambulatory activity.

### 2.5 Western immunoblotting

Western immunoblotting was employed to quantify the relative levels of pro-brain-derived neurotrophic factor (proBDNF) and mature BDNF proteins in the mouse hippocampus following treatment with CBD, ANI, and K252a. Previous research has demonstrated that hippocampal BDNF protein levels increase progressively within minutes after memory reactivation and reach a peak approximately 6 h post-reactivation (Radiske et al., 2015). Accordingly, bilateral hippocampal tissue samples were collected 6 h after memory reactivation and subsequent administration of CBD and K252a, in order to assess hippocampal BDNF protein expression during the reconsolidation phase of IA memory. Twenty mice were randomly assigned to four groups (*n* = 5 per group): Veh + Veh, Veh + K252a, CBD + Veh, and CBD + K252a. Bilateral hippocampal tissues were collected in microcentrifuge tubes and stored at −20 °C until further processing.

Previous findings by Kim et al. (2012) demonstrated that hippocampal BDNF protein levels increase between 0 and 6 h following IA acquisition, a time window that is critical for IA memory consolidation (Kim et al., 2012). Accordingly, to examine IA memory consolidation, mice were euthanized 6 h after IA training, following consecutive administrations of CBD, ANI, and K252a. This experimental design aimed to determine whether the effects of CBD and ANI co-treatment are mediated through the BDNF–TrkB signaling pathway in the hippocampus. Mice were randomly assigned to six experimental groups (*n* = 4 per group) before drug administration. Each group subsequently received the designated treatment according to the experimental protocol: Veh + Veh + Veh, CBD + Veh + Veh, Veh + ANI + Veh, Veh + ANI + K252a, CBD + ANI + Veh, and CBD + ANI + K252a. Bilateral hippocampal tissues were collected in microcentrifuge tubes and stored at −20 °C until further processing.

All hippocampal samples were homogenized in lysis buffer supplemented with a protease inhibitor cocktail. The homogenates were centrifuged at 13,500 rpm for 10 min at 4 °C. Protein concentrations were determined using the Bradford assay, with bovine serum albumin (BSA) as the standard. Equal amounts of protein (30 μg per sample) were denatured at 95 °C for 5 min in 2 × sample buffer containing 5% β-mercaptoethanol. Proteins were separated by 4%–12% SDS-PAGE and subsequently transferred onto PVDF membranes. Membranes were blocked with 5% non-fat milk in phosphate-buffered saline containing Tween 20 (PBST) for 1 h with gentle agitation. The membranes were then incubated overnight at 4 °C with a primary antibody against mature BDNF (14 kDa) and proBDNF (32 kDa) at a dilution of 1:1000 (Abcam, Cat# ab108319, RRID:AB_10862052). After three washes in PBST (total 30 min), membranes were incubated with a horseradish peroxidase-conjugated anti-rabbit secondary antibody (1:25000, Leadgene, Cat# LDG0014YE) for 1 h at room temperature, followed by additional washes. Protein bands were visualized using an enhanced chemiluminescence detection reagent and imaged with a fluorescence imaging system. Densitometric analysis was performed using ImageJ software. Protein expression levels were normalized against β-actin, which served as a loading control and was detected using a primary antibody (1:5000; Proteintech, Cat# 60008-1-Ig, RRID:AB_2289225) and a secondary antibody (1:25000; Leadgene, Cat# LDG0012YE). The Veh + Veh group and the Veh + Veh + Veh group were used as normalization controls for the reconsolidation and consolidation experiments, respectively.

Western blot images were acquired using an iBright FL1500 Imaging System (Invitrogen, Thermo Fisher Scientific, MA, USA, RRID:SCR_026331). Western blot signals were quantified using ImageJ software (ver. 1.54k, RRID:SCR_003070). Although densitometry was not performed in a blinded manner, all target bands across groups on the same transfer membrane were framed and quantified simultaneously to minimize potential rater bias. The geometric positions of the target bands were determined relative to the molecular weight protein marker. Band intensities were quantified using the built-in gel analysis function in ImageJ. The optic density of either proBDNF or mature BDNF was normalized to that of its corresponding loading control (β-actin). The Veh + Veh group in the reconsolidation experiment and the Veh + Veh + Veh group in the consolidation experiment were designated as the baseline groups, with their mean normalized densitometric values set to 100%. The normalized density of each individual band was then expressed as a percentage relative to the baseline group using the following formula: $R\,e\,l\,a\,t\,i\,v\,e\,L\,e\,v\,e\,l\,\frac{\mathrm{Normalized}\,{\mathrm{Densitometry}}_{\mathrm{Individual}\,\mathrm{Band}}}{\mathrm{Mean}\,\mathrm{of}\,\mathrm{Normalized}\,{\mathrm{Densitometry}}_{\mathrm{Baseline}\,\mathrm{Group}}}\,100\%$

### 2.6 Statistical analysis

Priori power analyses were conducted using G*Power software (version 3.1.9.7; RRID:SCR_013726) to determine the required sample sizes. Because biochemical assays typically exhibit lower variability than behavioral tests, a larger estimated effect size (Cohen’s *f* = 1.00) was selected for the assays, whereas a smaller effect size (Cohen’s *f* = 0.60) was set for the behavioral tests. For a one-way fixed-effects ANOVA with an alpha level of α = 0.05 and a statistical power of 1 - β = 0.80, the number of groups was determined by the experimental design. The results of power analyses indicated that minimum sample sizes of *n* = 4 and *n* = 9 per group were required for the Western blot assays and behavioral tests, respectively. Animals were randomly assigned to experimental groups, and no exclusion criteria were applied prior to or after behavioral testing. SPSS Statistics 17.0 was used for statistical analysis. The results of step-through latency and locomotion were presented as mean + standard error of the mean (SEM). Two-way repeated-measures ANOVA was applied to behavioral data because these experiments involved both between-subject factors (different drug treatment groups) and a within-subject factor (repeated testing across time points, i.e., Pretest, Test 1, and Test 2). In contrast, one-way ANOVA was used for biochemical analyses, as these data were obtained from a single time point and involved comparisons among multiple treatment groups without repeated measurements. *Post hoc* comparisons, when appropriate, were conducted using the Tukey HSD test or Student’s *t*-test. For comparisons corresponding to our predefined hypotheses (e.g., Figure 3C), planned contrasts were performed regardless of the significance of the omnibus ANOVA, consistent with recommendations for hypothesis-driven statistical testing (Rosenthal et al., 2000; Ruxton and Beauchamp, 2008). For all comparisons, the criterion for statistical significance was set as *p* < 0.05.

## 3 Results

### 3.1 CBD impaired IA memory reconsolidation

To determine whether CBD modulates IA memory reconsolidation, CBD or vehicle was administered immediately following memory reactivation (Figure 1A). Mice were randomly assigned to four groups: vehicle (Veh, *n* = 10) or CBD at doses of 5, 10, or 30 mg/kg (*n* = 10 per group). All animals successfully acquired IA memory, and no group differences were observed during the first retention test (Test 1), as confirmed by a two-way repeated-measures ANOVA (time, F_1,36_ = 251.195, *p* < 0.001; group, F_3,36_ = 0.034, *p* = 0.991; time × group interaction, F_3,36_ = 0.085, *p* = 0.968; Figure 1B). This was further supported by a one-way ANOVA showing no group differences prior to CBD administration (Test 1: F_3,36_ = 0.051, *p* = 0.985; Figure 1B). In contrast, a significant group effect emerged in the second retention test, as indicated by a one-way ANOVA (Test 2: F_3,36_ = 3.014, *p* = 0.043; Figure 1B). Notably, the effect was not statistically significant when analyzed with a non-parametric Kruskal-Wallis test (*p* = 0.059). *Post hoc* analyses revealed that mice treated with CBD at 10 mg/kg exhibited significantly reduced IA memory performance compared with both the Veh group (*p* = 0.01) and the CBD 5 mg/kg group (*p* = 0.032). No significant differences were observed for the 30 mg/kg group relative to controls. Importantly, CBD treatment did not affect locomotor activity at any dose (one-way ANOVA: F_3,36_ = 1.459, *p* = 0.242; Figure 1C), indicating that the observed effects on memory were not attributable to alterations in general activity levels. The unexpected inverted-U dose–response curve of CBD may be interpreted in the context of the Yerkes–Dodson law (Yerkes and Dodson, 1908) and is further discussed in the section “4 Discussion.” Together, these findings indicate that CBD dose-dependently impairs IA memory reconsolidation.

### 3.2 K252a reversed CBD-induced impairment of IA memory reconsolidation

To determine whether Trk receptor signaling mediates the effects of CBD on IA memory reconsolidation, we examined whether the Trk receptor antagonist K252a could reverse CBD-induced memory impairment. Mice were randomly assigned to four groups (*n* = 12 per group): Veh + Veh, Veh + K252a, CBD + Veh, and CBD + K252a. CBD (10 mg/kg) or Veh was administered immediately following memory reactivation, and K252a (5 μg/kg) or Veh was injected 30 min later (Figure 2A). All groups successfully acquired IA memory, with no significant differences observed during the first retention test (Test 1), as confirmed by a two-way repeated-measures ANOVA (time, F_1,44_ = 220.784, *p* < 0.001; group, F_3,44_ = 0.677, *p* = 0.571; time x group interaction, F_3,44_ = 0.427, *p* = 0.735; Figure 2B). A one-way ANOVA further confirmed the absence of group differences prior to drug administration (Test 1: F_3,44_ = 0.558, *p* = 0.646; Figure 2B). In contrast, a significant group effect was observed during the second retention test, as indicated by a one-way ANOVA (Test 2: F_3,44_ = 5.522, *p* = 0.003; Figure 2B). *Post hoc* analyses revealed that both the Veh + K252a and CBD + Veh groups exhibited significantly reduced IA memory performance compared with the Veh + Veh group (*p* = 0.002 and *p* = 0.001, respectively). Notably, the CBD + K252a group showed significantly higher IA memory retention than the CBD + Veh group (*p* = 0.049), indicating that K252a attenuated the impairing effect of CBD on memory reconsolidation. Importantly, neither CBD nor K252a, administered alone or in combination, affected locomotor activity (one-way ANOVA: F_3,36_ = 0.309, *p* = 0.819; Figure 2C), excluding non-specific effects on general activity. The mechanism underlying the paradoxical finding that K252a, which itself impairs memory, reverses the memory-impairing effects of CBD is discussed further in the section “4 Discussion.” Collectively, these findings indicate that both K252a and CBD alone impair IA memory reconsolidation, whereas K252a reverses the CBD-induced impairment, implicating Trk receptor signaling in this process.

### 3.3 CBD selectively reduced hippocampal proBDNF, but not mature BDNF, during IA memory reconsolidation

To investigate the molecular mechanisms underlying the effects of CBD and K252a on IA memory reconsolidation, we assessed whether these compounds modulate hippocampal BDNF and proBDNF protein levels. CBD (10 mg/kg) was administered immediately after memory reactivation, followed by K252a (5 μg/kg) 30 min later. Mice were sacrificed 6 h after CBD administration, and bilateral hippocampal tissues were collected for analysis (Figure 3A). Mice were randomly assigned to four groups (*n* = 5 per group): Veh + Veh, Veh + K252a, CBD + Veh, and CBD + K252a. Importantly, our primary hypothesis was specified as *a priori* based on previous evidence indicating that CBD facilitates fear-memory extinction through modulation of BDNF signaling. Specifically, before data collection, we hypothesized that CBD administration during the reconsolidation window would modulate hippocampal BDNF or proBDNF levels relative to the corresponding vehicle-treated control (CBD + Veh vs. Veh + Veh). Consistent with this predefined hypothesis, a planned contrast revealed a significant reduction in hippocampal proBDNF levels in the CBD + Veh group compared with the Veh + Veh group (independent *t*-test, *p* = 0.019; Figure 3C). In contrast, hippocampal BDNF levels did not differ significantly between the two groups (independent *t*-test, *p* = 0.415; Figure 3C). Moreover, mice receiving combined CBD and K252a treatment (CBD + K252a) exhibited proBDNF levels that did not significantly differ from those observed in the Veh + Veh (*p* = 0.546), Veh + K252a (*p* = 0.258), and CBD + Veh (*p* = 0.318) groups (Figure 3C). Because this comparison was specified as *a priori*, it represents a planned contrast rather than an exploratory *post hoc* comparison. Planned contrasts are appropriate for testing a limited number of hypothesis-driven comparisons and do not require a statistically significant omnibus ANOVA when the comparisons are defined before data collection (Rosenthal et al., 2000; Ruxton and Beauchamp, 2008). It is important to note that this preliminary observation should be interpreted with caution and warrants further replication in future studies. Quantitative analysis, however, revealed no significant differences among the four groups in hippocampal mature BDNF (14 kDa) levels (one-way ANOVA: F_3,16_ = 0.582, *p* = 0.635; Figure 3B). Similarly, no significant group differences were observed in proBDNF (32 kDa) levels (one-way ANOVA: F_3,16_ = 1.783, *p* = 0.191; Figure 3C). Collectively, these findings indicate that CBD selectively reduces hippocampal proBDNF levels, an effect that is partially reversed by K252a, when compared with the Veh + Veh group. These results suggest that modulation of hippocampal proBDNF signaling may contribute to the impairing effect of CBD on IA memory reconsolidation.

### 3.4 CBD did not impair IA memory consolidation in contrast to ANI

To examine whether CBD affects IA memory consolidation, CBD or Veh was administered immediately after IA training (Figure 4A). Mice were randomly assigned to receive CBD (5, 10, or 30 mg/kg; *n* = 13 per group) or Veh (*n* = 13). All groups acquired IA memory, as indicated by a significant main effect of time (two-way repeated-measures ANOVA: time, F_1,48_ = 179.596, *p* < 0.001), with no significant effect of group (F_3,48_ = 0.375, *p* = 0.771) or time × group interaction (F_3,48_ = 0.219, *p* = 0.883) (Figure 4B). When CBD was administered immediately following IA training, however, no significant differences were observed among groups during the retention test (one-way ANOVA: F_3,48_ = 0.29, *p* = 0.833; Figure 4B). In addition, CBD did not affect locomotor activity (one-way ANOVA: F_3,36_ = 0.536, *p* = 0.661; Figure 4D). These findings indicate that CBD does not affect IA memory consolidation under the present conditions.

To verify that the experimental parameters were sufficient to detect consolidation deficits, ANI was used as a positive control. ANI or Veh was administered immediately after IA training (Figure 4A), and mice were assigned to ANI (50 or 150 mg/kg; *n* = 12 per group) or Veh (*n* = 12) groups. All mice successfully acquired IA memory; however, following ANI administration, there were significant effects of time (two-way repeated-measures ANOVA: F_1,33_ = 57.223, *p* < 0.001), group (F_2,33_ = 5.169, *p* = 0.011), and a significant time × group interaction (F_2,33_ = 5.02, *p* = 0.012) (Figure 4C). Analysis of simple main effects revealed a significant group difference following ANI treatment (F_2,33_ = 5.128, *p* = 0.012). *Post hoc* tests showed that the ANI-150 group exhibited significantly reduced IA memory retention compared with the control group (*p* = 0.009) (Figure 4C). Locomotor activity was not affected by ANI treatment (one-way ANOVA: F_2,27_ = 0.151, *p* = 0.861; Figure 4E). Together, these results demonstrate that ANI impairs IA memory consolidation, consistent with previous reports (Monleón Verdú et al., 2008; Zhang et al., 2011).

### 3.5 CBD reversed ANI-induced impairment of IA memory consolidation

We next examined whether CBD (10 mg/kg) could reverse the impairing effect of ANI (150 mg/kg) on IA memory consolidation. Mice were randomly assigned to four groups (*n* = 16 per group): Veh + Veh, CBD + Veh, Veh + ANI, and CBD + ANI. CBD and ANI (or their respective vehicles) were administered sequentially following IA training (Figure 5A). All mice successfully acquired IA memory, as indicated in the test session (Figure 5B). A two-way repeated-measures ANOVA revealed significant effects of time (F_1,60_ = 211.715, *p* < 0.001), group (F_3,60_ = 6.546, *p* = 0.001), and a significant time × group interaction (F_3,60_ = 6.189, *p* = 0.001). Simple main effects analysis indicated that group differences were significant only during the test phase following drug administration (F_3,60_ = 6.4, *p* = 0.001). *Post hoc* analyses revealed that the Veh + ANI group exhibited significantly reduced IA memory retention compared with both the Veh + Veh (*p* = 0.019) and CBD + ANI (*p* = 0.001) groups, indicating that CBD effectively reversed the impairing effect of ANI on IA memory consolidation (Figure 5B). Finally, neither CBD nor ANI, whether administered alone or in combination, affected locomotor activity, as confirmed by a one-way ANOVA showing no significant group effect (F_3,36_ = 0.469, *p* = 0.706; Figure 5C).

### 3.6 K252a blocked CBD-mediated rescue of ANI-induced impairment in IA memory consolidation

We used K252a to determine whether the BDNF–TrkB signaling pathway mediates CBD’s rescue of ANI-induced impairment in IA memory consolidation. CBD (10 mg/kg) and ANI (150 mg/kg), or their respective Veh, were administered consecutively immediately after IA training, followed by K252a (5 μg/kg) or Veh injection 30 min later (Figure 6A). Mice were randomly assigned to four groups (*n* = 13 per group): Veh + ANI + Veh, Veh + ANI + K252a, CBD + ANI + Veh, and CBD + ANI + K252a. All groups successfully acquired IA memory during training (Figure 6B). Two-way repeated-measures ANOVA revealed significant effects of time (F_1,48_ = 138.968, *p* < 0.001), group (F_3,48_ = 4.44, *p* = 0.008), and a significant time × group interaction (F_3,48_ = 5.064, *p* = 0.004). Simple main effects analysis showed that group differences were significant only during the retention test (F_3,48_ = 4.76, *p* = 0.006) (Figure 6B). *Post hoc* tests demonstrated that the CBD + ANI + Veh group exhibited significantly higher IA memory scores than the Veh + ANI + Veh group (*p* = 0.013), indicating that CBD reversed the memory-impairing effect of ANI. Importantly, the CBD + ANI + K252a group showed significantly lower IA memory scores than the CBD + ANI + Veh group (*p* = 0.015), indicating that K252a blocked the rescuing effect of CBD on ANI-induced impairment of IA memory consolidation. Finally, none of the drug treatments significantly affected locomotor activity (one-way ANOVA: F_3,36_ = 0.234, *p* = 0.872; Figure 6C).

### 3.7 K252a blocked the rescuing effect of CBD on ANI-induced reductions in hippocampal BDNF levels during IA memory consolidation

**FIGURE 7:**
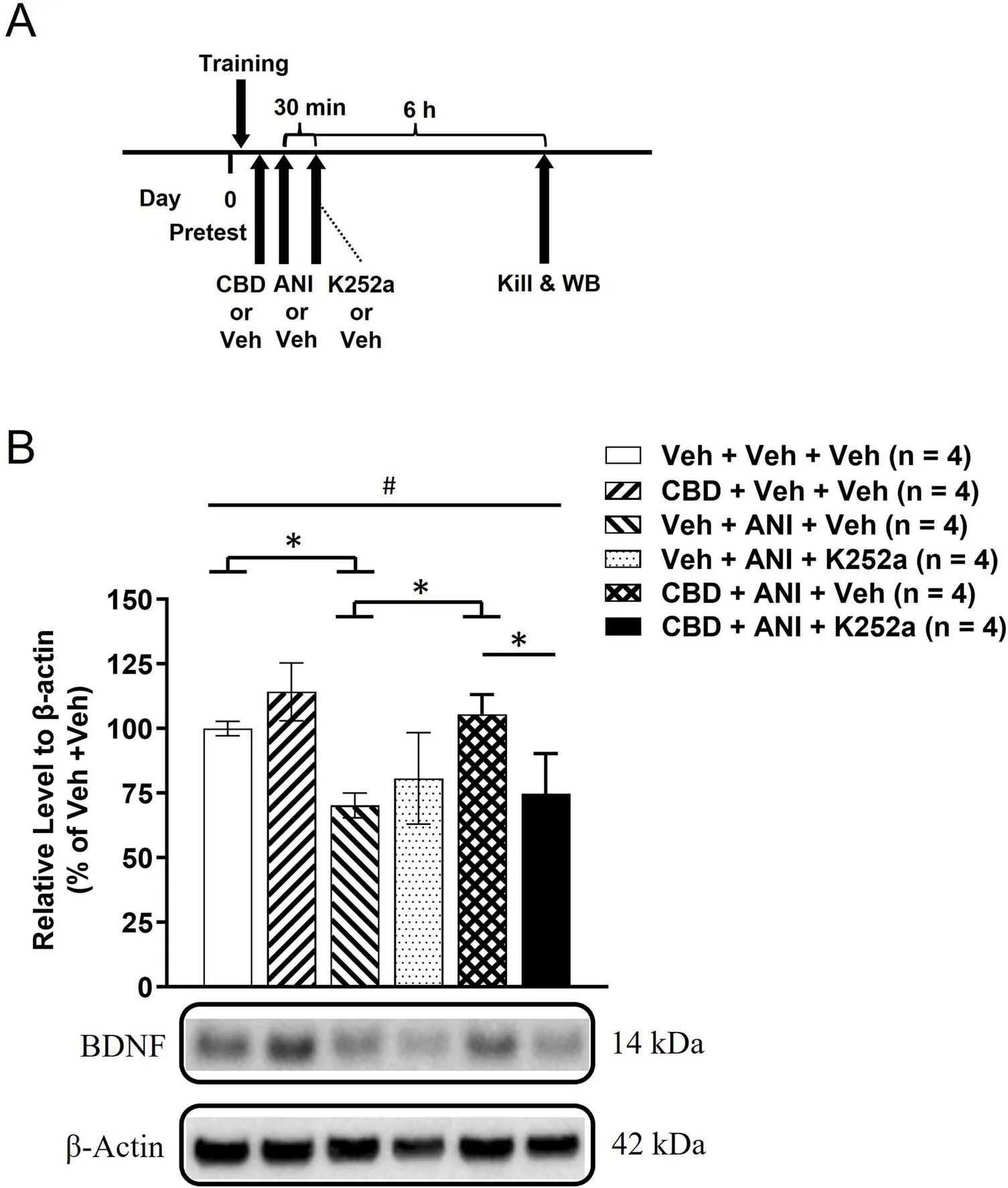
Cannabidiol (CBD) reversed ANI-induced decreases in hippocampal mature BDNF protein levels, an effect blocked by K252a. **(A)** Experimental timeline illustrating the effects of CBD (10 mg/kg), ANI (150 mg/kg), and K252a (5 μg/kg) on hippocampal BDNF protein expression at 6 h after IA training. Mice were randomly assigned to six groups (*n* = 4 per group): Veh + Veh + Veh, CBD + Veh + Veh, Veh + ANI + Veh, Veh + ANI + K252a, CBD + ANI + Veh, and CBD + ANI + K252a. **(B)** A one-way ANOVA revealed a significant effect of drug treatment on hippocampal mature BDNF (14 kDa) protein levels (F_5,18_ = 3.597, ^#^*p* < 0.05). *Post hoc* analyses showed that CBD-alone (CBD + Veh + Veh) did not alter hippocampal mature BDNF protein levels at 6 h after IA training (*p* > 0.05), whereas ANI-alone (Veh + ANI + Veh) significantly reduced mature BDNF expression (*p < 0.05). No significant difference in hippocampal mature BDNF expression was observed between the ANI-alone (Veh + ANI + Veh) and ANI-plus-K252a (Veh + ANI + K252a) groups (*p* > 0.05). CBD treatment reversed the ANI-induced decrease in hippocampal mature BDNF protein levels (*p < 0.05), and this rescuing effect was blocked by K252a administration (*p < 0.05).

To investigate the molecular mechanisms underlying the effects of ANI, CBD, and K252a on IA memory consolidation, CBD (10 mg/kg) and ANI (150 mg/kg) or their respective Veh were administered consecutively immediately after IA training, followed by injection of K252a (5 μg/kg) or Veh 30 min later. The brains were dissected, and bilateral hippocampal tissues were collected 6 h after ANI administration (Figure 7A). Twenty-four mice were randomly assigned to six groups (*n* = 4 per group): Veh + Veh + Veh, CBD + Veh + Veh, Veh + ANI + Veh, Veh + ANI + K252a, CBD + ANI + Veh, and CBD + ANI + K252a. A one-way ANOVA revealed a significant effect of drug treatment on hippocampal mature BDNF (14 kDa) protein levels (F_5,18_ = 3.597, *p* = 0.02) (Figure 7B). *Post hoc* analyses showed that the CBD-alone group (CBD + Veh + Veh) did not significantly change hippocampal mature BDNF levels compared with the control group (Veh + Veh + Veh; *p* = 0.305). In contrast, the ANI-alone group (Veh + ANI + Veh) significantly reduced mature BDNF protein expression in the hippocampus relative to the control group (*p* = 0.04) (Figure 7B). No significant difference in hippocampal mature BDNF expression was observed between the ANI-alone (Veh + ANI + Veh) and ANI-plus-K252a (Veh + ANI + K252a) groups (*p* = 0.446). Compared with the ANI-alone group, CBD-plus-ANI (CBD + ANI + Veh) significantly reversed mature BDNF protein levels in the hippocampus (*p* = 0.017). Importantly, administration of the TrkB antagonist K252a 30 min after consecutive CBD and ANI treatment (CBD + ANI + K252a) significantly reduced hippocampal mature BDNF levels compared with the CBD + ANI + Veh group (*p* = 0.035) (Figure 7B). Collectively, these findings suggest that BDNF–TrkB signaling contributes to CBD-mediated rescue of ANI-induced impairment in IA memory consolidation.

## 4 Discussion

The primary objective of this study was to investigate the effects of CBD on IA memory reconsolidation and consolidation processes. We first demonstrated that CBD impaired IA memory reconsolidation in a dose-dependent manner, and this effect was reversed by the Trk receptor antagonist K252a. Biochemical analyses further revealed that the disruptive effect of CBD on IA memory reconsolidation was associated with a reduction in hippocampal proBDNF protein levels, without significant alterations in mature BDNF expression, when compared with the Veh + Veh group. Notably, the effective dose of CBD (10 mg/kg) that impaired reconsolidation did not influence IA memory consolidation. Instead, this dose of CBD attenuated ANI-induced impairment of IA memory consolidation, an effect that was abolished by K252a. Consistent with these behavioral findings, biochemical analyses showed that ANI reduced hippocampal mature BDNF levels, whereas CBD treatment reversed this reduction. This rescuing effect of CBD on mature BDNF expression was also blocked by K252a. Taken together, these findings suggest that CBD differentially modulates IA memory reconsolidation and consolidation that may be associated with distinct BDNF–TrkB signaling mechanisms in the mouse hippocampus.

To the best of our knowledge, this is the first study to systematically examine the effects of CBD on both IA memory reconsolidation and consolidation processes. The null effects of CBD on IA memory consolidation appear to contrast with its impairing effects in the CFC paradigm. Stern et al. (2017) demonstrated that CBD disrupts CFC memory consolidation, as evidenced by reduced freezing behavior when rats were re-exposed either to the conditioned context in the absence of footshock (specific consolidation) or to a novel, unpaired context (generalized consolidation). Specifically, CBD (10 mg/kg) impaired specific memory consolidation, whereas higher doses (10 and 30 mg/kg) disrupted generalized memory consolidation of CFC (Stern et al., 2017). In contrast, the effects of CBD on IA memory consolidation remain inconsistent, with studies reporting either no effect (Fagherazzi et al., 2012) or enhancement of memory consolidation (Kruk-Slomka and Biala, 2021). Notably, subeffective doses of CBD have also been shown to reverse memory impairments induced by MK-801 and scopolamine in the IA paradigm (Kruk-Slomka and Biala, 2021; Kruk-Slomka et al., 2024).

We propose that these seemingly discrepant findings may be attributable to fundamental differences between the CFC paradigm and the IA task, underscoring the importance of delineating the distinct behavioral and neurobiological characteristics underlying these two paradigms. The CFC paradigm, grounded in classical conditioning, involves the association between a neutral context and an aversive stimulus, eliciting freezing behavior as a passive defensive response and modeling uncontrollable stress. In contrast, the IA task incorporates both classical and operant components, requiring subjects to actively suppress its innate preference for the dark compartment in favor of avoiding an aversive outcome, thereby reflecting goal-directed learning and adaptive coping strategies (Liang, 2009). These behavioral differences are paralleled by distinct neural mechanisms. While the amygdala is essential for acquisition but less involved in consolidation in CFC, it contributes to consolidation in IA (Wilensky et al., 2000). Moreover, the nucleus accumbens (NAc) is not critical for norepinephrine-induced memory enhancement in CFC but is required in IA, which engages a broader dorsal hippocampus (DH)–medial prefrontal cortex (mPFC)–NAc circuit associated with active coping (Yang and Liang, 2014). Although the DH is involved in both paradigms, it primarily supports contextual encoding and retrieval in CFC (Fanselow, 2000; Maren et al., 2013; Wiltgen et al., 2004), whereas it is preferentially engaged during early consolidation in IA (Izquierdo et al., 1997; Roozendaal and McGaugh, 2011). Collectively, these distinctions indicate that the IA task constitutes a more appropriate model for investigating goal-directed learning and active avoidance behavior in fear-related memory processes, and further suggest that elucidating hippocampal mechanisms within this paradigm may yield critical insights into the effects of CBD on memory-associated disorders, including post-traumatic stress disorder (PTSD) and amnesia (Liang, 2009).

In addition to its effects on consolidation, CBD has also been shown to disrupt memory reconsolidation in the CFC paradigm. Specifically, systemic administration of CBD immediately following memory reactivation decreases freezing behavior, an effect that appears to be mediated, at least in part, by CB_1_ receptor activation (Gazarini et al., 2014; Stern et al., 2012). To date, no study had examined the effects of CBD on IA memory reconsolidation. Our findings provide the first evidence that CBD impairs IA memory reconsolidation, an effect that is abolished by the Trk receptor antagonist K252a and is accompanied by a reduction in hippocampal proBDNF protein levels. CBD impaired IA memory reconsolidation in a dose-dependent manner, with a significant effect observed at 10 mg/kg but not at 5 or 30 mg/kg. This unexpected inverted U-shaped dose–response relationship resembles the bidirectional effects of post-training administration of epinephrine, norepinephrine, adrenocorticotropic hormone (ACTH), or amygdala stimulation on IA memory consolidation (Gold et al., 1975; Gold and Van Buskirk, 1975, 1976). Such a pattern may be interpreted within the framework of the Yerkes–Dodson law, which posits that optimal memory performance occurs at intermediate levels of physiological or neural activation, whereas both lower and higher levels are less effective (Yerkes and Dodson, 1908). Our finding is consistent with prior evidence demonstrating that CBD dose-dependently disrupts memory reconsolidation in the CFC paradigm, with 10 mg/kg likewise reported as the most effective dose (Stern et al., 2012).

A notable finding of the present study is that K252a alone impaired IA memory reconsolidation, whereas co-administration of K252a attenuated the disruptive effect of CBD. The impairment produced by K252a is consistent with previous evidence demonstrating that pharmacological inhibition of TrkB signaling disrupts hippocampus-dependent memory reconsolidation and synaptic plasticity (Lu et al., 2008; Minichiello, 2009). Likewise, intrahippocampal and intraperitoneal administration of the TrkB antagonists K252a or ANA-12 has been reported to impair IA memory reconsolidation (Blank et al., 2016; Huang et al., 2021; Liu et al., 2008). The observation that K252a attenuated, rather than enhanced, the disruptive effect of CBD suggests that the interaction between CBD and BDNF–TrkB signaling is unlikely to reflect a simple additive pharmacological effect. Instead, these findings may indicate that CBD and TrkB-dependent signaling converge on overlapping molecular pathways regulating memory reconsolidation, such that pharmacological inhibition of one component alters the net behavioral outcome. Similar state-dependent roles of BDNF–TrkB signaling in memory destabilization, reconsolidation, and synaptic plasticity have been reported previously (Andero et al., 2014; Rattiner et al., 2004), suggesting that the functional consequences of TrkB signaling depend on the timing, brain region, and state of the memory trace.

However, this interpretation should be considered preliminary. The present study did not directly examine TrkB phosphorylation or downstream signaling pathways, including ERK1/2, Akt, or PLCγ activation, nor was a dose-response analysis performed for K252a. Furthermore, K252a is known to inhibit multiple protein kinases in addition to Trk receptors (Knüsel and Hefti, 1992; Tapley et al., 1992), precluding definitive attribution of the observed behavioral effects solely to TrkB inhibition. Accordingly, the present findings support the involvement of BDNF–TrkB-related signaling in CBD-induced modulation of memory reconsolidation but do not establish the underlying molecular mechanisms. Future studies using selective TrkB antagonists, direct assessment of TrkB phosphorylation and downstream signaling, and complementary genetic approaches will be necessary to elucidate these mechanisms.

It is important to note that our biochemical findings are interpreted exclusively as relative differences among the experimental treatment groups, with the Veh + Veh group serving as the reference condition. Specifically, relative to the Veh + Veh group, CBD treatment was associated with reduced hippocampal proBDNF expression without significantly affecting hippocampal mature BDNF levels. In contrast, K252a alone affected neither proBDNF nor mature BDNF levels, despite disrupting memory reconsolidation. To our knowledge, only one previous study has examined the relationship between CBD and proBDNF, reporting that chronic CBD treatment increased cortical proBDNF levels without affecting recognition memory (Watt et al., 2026). Thus, the involvement of proBDNF signaling in CBD-induced memory impairment remains largely unexplored. Notably, K252a partially attenuated the CBD-induced reduction in proBDNF while rescuing memory performance, suggesting a dissociation between behavioral and biochemical outcomes. Because K252a primarily inhibits Trk receptor activation and may also affect other plasticity-related kinases, including PKC and CaMKs (Kase et al., 1986), its effects are unlikely to be attributable solely to TrkB blockade. Furthermore, proBDNF can signal through both p75NTR and, under certain conditions, TrkB receptors, and K252a may indirectly influence proBDNF processing through modulation of tPA activity (Hwang et al., 2011). Consequently, the observed interaction likely reflects the involvement of multiple neurotrophin-dependent signaling pathways. An additional limitation of the present study is that the antibody employed detected both mature BDNF and proBDNF, which may limit the precision of proBDNF quantification. Consequently, although our findings suggest that CBD-induced impairment of IA memory reconsolidation is associated with reduced hippocampal proBDNF levels, the relative contributions of proBDNF–TrkB and proBDNF–p75NTR signaling remain unclear. Future studies employing isoform-specific antibodies, receptor-selective pharmacological tools, and direct assessments of downstream signaling pathways will be required to delineate the respective roles of these signaling mechanisms.

Another limitation of the present study is the absence of a naïve (no-training) control group. Consequently, our findings cannot determine whether the observed hippocampal BDNF and proBDNF levels represent basal physiological expression or reflect changes induced by IA training and/or memory reactivation. However, basal hippocampal BDNF expression has been characterized previously. For example, Kim et al. (2012) included a naïve control group and quantified hippocampal BDNF levels to establish baseline expression for comparison with learning-induced changes (Kim et al., 2012). In the present study, all experimental groups underwent identical behavioral procedures and differed only in pharmacological treatment. Therefore, the observed molecular differences can be interpreted as treatment-related effects within the context of memory reconsolidation. Nevertheless, future studies incorporating both naïve and non-reactivated control groups will be important for distinguishing basal neurotrophin expression from molecular alterations associated with learning and memory reconsolidation.

Accumulating evidence indicates that proBDNF is converted into mature BDNF in the rat hippocampus following memory reactivation in both the object recognition and IA tasks (Radiske et al., 2015, 2017). Temporally, proBDNF levels increase within 5 min to 1.5 h after IA memory reactivation and subsequently return to baseline by 3–6 h. In contrast, mature BDNF levels remain relatively stable during the early post-reactivation period (5 min to 1.5 h) and increase at later time points (3–6 h). Pharmacological inhibition of the conversion of proBDNF to mature BDNF in the hippocampus has been shown to facilitate extinction of CFC memory, while impairing CFC memory formation (Barnes and Thomas, 2008). These findings suggest that a dynamic balance between proBDNF and mature BDNF is critical for both memory reconsolidation and consolidation processes. In line with this framework, our findings demonstrate that CBD reduces hippocampal proBDNF levels, thereby impairing IA memory reconsolidation, whereas ANI decreases hippocampal BDNF levels, leading to deficits in IA memory consolidation. These preclinical observations are supported by clinical evidence. For instance, the parietal cortex of patients with Alzheimer’s disease exhibits an approximately 40% reduction in proBDNF protein levels compared to controls (Michalski and Fahnestock, 2003). Moreover, the basal nucleus of Meynert, a key structure implicated in Alzheimer’s disease pathology, shows a ∼50% decrease in BDNF mRNA expression (Fahnestock et al., 2002). Consistently, reductions in both proBDNF and BDNF levels in the parietal cortex have been positively correlated with cognitive decline (Peng et al., 2005), suggesting that both forms are essential for maintaining synaptic and cellular integrity underlying cognitive function. Taken together, these findings indicate that the balance between proBDNF and mature BDNF is a critical determinant of memory formation and reconsolidation. Elucidating the molecular mechanisms governing the conversion of proBDNF to mature BDNF may therefore provide valuable insights into potential therapeutic strategies for Alzheimer’s disease (Borodinova and Salozhin, 2017).

Interestingly, CBD (10 mg/kg) alone did not affect IA memory consolidation but effectively reversed ANI-induced consolidation deficits. This restorative effect was abolished by co-administration of the Trk receptor antagonist K252a, indicating the involvement of Trk-dependent signaling. Consistent with these behavioral findings, biochemical analyses revealed that, relative to the Veh + Veh group, ANI treatment was associated with reduced hippocampal mature BDNF levels, whereas CBD treatment attenuated this reduction. These results agree with previous studies showing that CBD reverses memory impairments induced by MK-801, scopolamine, sepsis, pneumococcal meningitis, and other neurological conditions (Barichello et al., 2012; Cassol et al., 2010; Kruk-Slomka and Biala, 2021; Kruk-Slomka et al., 2024; Patra et al., 2019; Peres et al., 2016). Collectively, our findings suggest that CBD differentially regulates IA memory processes, impairing reconsolidation while restoring ANI-disrupted consolidation. Notably, these distinct behavioral effects were accompanied by differential regulation of hippocampal neurotrophins, with CBD-induced reconsolidation impairment associated with reduced proBDNF levels and CBD-mediated rescue of consolidation associated with restoration of mature BDNF expression.

BDNF is a key regulator of hippocampal synaptic plasticity and memory consolidation. Through activation of TrkB receptors and downstream PI3K/Akt, MAPK/ERK, and PLCγ signaling pathways, BDNF promotes synaptic strengthening, dendritic spine remodeling, protein synthesis, and CREB-dependent transcription required for long-term memory formation (Lu et al., 2008; Minichiello, 2009). BDNF–TrkB signaling is also essential for the induction and maintenance of hippocampal long-term potentiation, a major cellular substrate of memory consolidation. Therefore, the restoration of hippocampal BDNF by CBD may counteract ANI-induced consolidation deficits by re-establishing neurotrophin-dependent synaptic plasticity and stabilizing memory-related neuronal circuits. Although downstream signaling events and electrophysiological correlates were not examined in the present study, the blockade of CBD’s beneficial effect by K252a supports the involvement of BDNF–TrkB signaling in this process.

A parsimonious explanation for the differential effects of CBD on memory consolidation and reconsolidation is that these processes are mechanistically distinct. Reconsolidation is induced by memory reactivation and is generally more labile than consolidation, rendering it more susceptible to pharmacological disruption (Anokhin et al., 2002; McGaugh, 2000; McKenzie and Eichenbaum, 2011). Consistent with this view, CBD selectively impaired IA memory reconsolidation at 10 mg/kg but did not affect consolidation, even at doses up to 30 mg/kg. Although consolidation and reconsolidation were traditionally thought to share common molecular mechanisms, including MAPK/ERK, PKA, and CREB signaling (Kelly et al., 2003; Kida et al., 2002; Koh and Bernstein, 2003), accumulating evidence indicates that they engage distinct neural circuits, temporal dynamics, and molecular pathways (Alberini, 2005; Debiec et al., 2002; McKenzie and Eichenbaum, 2011; Miller and Sweatt, 2006; Riccio and Cullen, 2012). For example, BDNF is required for consolidation but not reconsolidation in contextual fear conditioning, whereas Zif268 shows the opposite pattern (Lee et al., 2004; Lee and Hynds, 2013). This distinction is consistent with our findings that hippocampal BDNF is not required for CBD-induced disruption of IA memory reconsolidation, but is critically involved in the restorative effect of CBD on memory consolidation, further supporting the view that consolidation and reconsolidation are dissociable memory processes.

Previous studies have demonstrated that CBD modulates a range of cognitive functions through alterations in BDNF protein expression across distinct brain regions, with effects that depend on dose, treatment regimen, and behavioral paradigm. For instance, CBD administration 30 min or 7 days prior to the forced swim test produces antidepressant-like effects; however, only acute treatment increases BDNF protein levels in both the prefrontal cortex (PFC) and hippocampus of Swiss mice (Sales et al., 2019). In contrast, chronic CBD administration has been reported to decrease BDNF expression in the frontal cortex and hippocampus, but not in the striatum, accompanied by enhanced fear expression in the conditioned emotional response test (ElBatsh et al., 2012). Moreover, chronic CBD treatment administered 1 h after predator exposure does not alter BDNF protein levels in the frontal cortex, hippocampus, or amygdala in rats (Campos et al., 2012). Our results, however, indicate that acute CBD administration following IA training (consolidation) or memory reactivation (reconsolidation) does not affect hippocampal BDNF levels when assessed 6 h post-treatment. Collectively, these observations suggest that CBD-induced changes in BDNF expression are time-dependent and may be transient, becoming undetectable at later time points.

Specifically, relative to the Veh + Veh group, CBD treatment did not significantly alter total hippocampal mature BDNF levels but was associated with a significant reduction in hippocampal proBDNF expression following memory reactivation. At first glance, the association between reduced proBDNF and impaired reconsolidation appears paradoxical, as proBDNF is generally considered a negative regulator of synaptic plasticity through p75NTR-mediated signaling that promotes long-term depression and synaptic weakening (Borodinova and Salozhin, 2017; Lu et al., 2005). Consistent with this view, increased proBDNF signaling has been associated with impaired memory performance and suppression of conditioned fear memory (Buhusi et al., 2017; Sun et al., 2018; Wong et al., 2019). However, the role of proBDNF in reconsolidation may differ from its role in memory formation. Reconsolidation requires an initial destabilization phase following memory retrieval, during which the memory trace becomes labile before restabilization (Lee, 2009; Nader and Hardt, 2009). Given that proBDNF–p75NTR signaling promotes synaptic weakening (Lu et al., 2005), reduced proBDNF levels may limit the destabilization processes necessary for successful reconsolidation. Thus, CBD-induced reductions in proBDNF may impair memory updating by preventing adequate memory destabilization. Although this interpretation remains speculative, it offers a potential explanation for the seemingly paradoxical relationship between reduced proBDNF expression and impaired reconsolidation observed in the present study.

It is well established that IA memory consolidation requires *de novo* protein synthesis (Taubenfeld et al., 2001; Vianna et al., 2001). We found that administration of the protein synthesis inhibitor ANI dose-dependently disrupted IA memory consolidation, thereby replicating prior reports of ANI-induced impairment of IA memory consolidation (Monleón Verdú et al., 2008; Zhang et al., 2011). We hypothesize that this impairment may be mediated, at least in part, by alterations in hippocampal BDNF expression. Supporting this notion, previous studies have shown that microinjection of anti-BDNF antibodies into the hippocampus impairs IA memory, whereas infusion of recombinant human BDNF enhances aversive memory retention (Alonso et al., 2002). Moreover, intrahippocampal administration of BDNF has been reported to reverse ANI-induced deficits in IA memory retention (Bekinschtein et al., 2008a). In line with these findings, our results demonstrate that ANI reduces hippocampal BDNF protein levels, which may underlie its impairing effect on IA memory consolidation. Notably, CBD administered immediately after IA training did not alter mature BDNF levels under baseline conditions. However, CBD effectively reversed the ANI-induced reduction in hippocampal BDNF expression, an effect that was abolished by the TrkB receptor inhibitor K252a. This biochemical evidence closely parallels our behavioral observations, in which CBD attenuated ANI-induced impairments in IA memory consolidation, with this protective effect similarly blocked by K252a. Taken together, these findings suggest that CBD mitigates ANI-induced memory deficits, an effect that may be associated with the attenuation of ANI-induced reductions in hippocampal BDNF levels. Thus, the restorative effect of CBD on IA memory consolidation may be associated with activation of the BDNF–TrkB signaling pathway.

It is important to note that only male C57BL/6J mice were included in the present study, which limits the generalizability of our findings. Male rodents were selected in behavioral neuroscience studies because this has historically been considered a strategy to minimize variability associated with fluctuations in ovarian hormones across the estrous cycle, which may influence behavioral performance, stress responsiveness, and pharmacological sensitivity (Becker et al., 2005). Although recent evidence suggests that behavioral variability in female rodents is not necessarily greater than that observed in males (Becker et al., 2016; Prendergast et al., 2014), female animals remain underrepresented in preclinical neuroscience research. Accumulating evidence further suggests that sex may influence CBD pharmacokinetics and neurobehavioral effects (Matheson et al., 2022), and sex differences have also been reported in fear memory processing, BDNF-related signaling, and the neurobiology of PTSD (Bauer, 2023; Notaras and van den Buuse, 2020). However, the available literature on sex-specific effects of CBD remains limited and, in some cases, inconsistent. Therefore, it remains unclear whether the effects observed in male mice would extend to females. Future studies including both sexes are needed to determine the extent to which biological sex influences the effects of CBD on ANI-induced memory impairment and its associated neurobiological mechanisms.

In summary, our findings indicate that CBD differentially modulates IA memory consolidation and reconsolidation. CBD disrupts memory reconsolidation, suggesting potential utility for attenuating maladaptive aversive memories. In contrast, CBD does not impair IA memory consolidation under baseline conditions and can reverse pharmacologically induced memory deficits, indicating potential relevance for memory-related disorders. However, several limitations should be considered. Although systemic administration is clinically relevant and practically advantageous, it precludes definitive conclusions regarding region-specific mechanisms and causal relationships between CBD’s behavioral effects and hippocampal BDNF signaling. In addition, whether the effects of systemic ANI on IA memory consolidation reflect specific memory processes or non-specific physiological influences remains unclear. Future studies using site-specific hippocampal manipulations of CBD and ANI are therefore required. Moreover, the potential involvement of the amygdala in CBD-mediated modulation of IA memory remains insufficiently explored, despite limited evidence of reduced amygdala activity following CBD administration (Todd and Arnold, 2016). Finally, the mechanisms underlying the differential involvement of proBDNF and BDNF signaling in CBD’s effects on reconsolidation and consolidation remain unresolved. Elucidating the molecular switch between proBDNF and mature BDNF signaling will be essential for understanding CBD’s neurobiological actions and may provide therapeutic insights for memory-related disorders.

## Data Availability

The raw data supporting the conclusions of this article will be made available by the authors, without undue reservation.
